# Uncovering the Characteristics of Pupil Cycle Time (PCT) in Neuropathies and Retinopathies

**DOI:** 10.3390/vision9030051

**Published:** 2025-06-30

**Authors:** Laure Trinquet, Suzon Ajasse, Frédéric Chavane, Richard Legras, Frédéric Matonti, José-Alain Sahel, Catherine Vignal-Clermont, Jean Lorenceau

**Affiliations:** 1 Faculté des Sciences Médicales et Paramédicales, Aix-Marseille Université, 13385 Marseille, France; laure.trinquet@univ-amu.fr; 2 Institut de la Vision, Sorbonne Université, INSERM, CNRS, 75252 Paris, France; 3 Institut des Neurosciences de la Timone-CNRS UMR 7289, 13005 Marseille, France; frederic.chavane@univ-amu.fr; 4 LuMIn, CNRS, ENS Paris-Saclay, Centrale Supelec, Université Paris-Saclay, 91190 Orsay, France; richard.legras@ens-paris-saclay.fr; 5 Centre Monticelli Paradis d’Ophtalmologie, 13008 Marseille, France; frederic.matonti@free.fr; 6 Department of Ophthalmology, University of Pittsburgh, Pittsburgh, PA 15219, USA; sahelja@upmc.edu; 7 Hopital Fondation, Adolphe de Rothschild 29, Rue Manin, 75019 Paris, France; cvignal@for.paris; 8 Integrative Neuroscience and Cognition Center, UMR8002, Université Paris Cité, 75006 Paris, France

**Keywords:** pupil, oscillation, diseases, retina

## Abstract

Pupil cycle time (PCT) estimates the dynamics of a biofeedback loop established between pupil size and stimulus luminance, size or colour. The PCT is useful for probing the functional integrity of the retinopupillary circuits, and is therefore potentially applicable for assessing the effects of damage due to retinopathies or neuropathies. In previous studies, PCT was measured by manually counting the number of pupil oscillations during a fixed period to calculate the PCT. This method is scarce, requires a good expertise and cannot be used to estimate several PCT parameters, such as the oscillation amplitude or variability. We have developed a computerised setup based on eye-tracking that expands the possibilities of characterising PCT along several dimensions: oscillation frequency and regularity, amplitude and variability, which can be used with a large palette of stimuli (different colours, sizes, shapes or locations), and further allows measuring blinking frequency and eye movements. We used this method to characterise the PCT in young control participants as well as in patients with several pathologies, including age-related macular degeneration (AMD), diabetic retinopathy (DR), retinitis pigmentosa (RP), Stargardt disease (SD), and Leber hereditary optic neuropathy (LHON). We found that PCT is very regular and stable in young healthy participants, with little inter-individual variability. In contrast, several PCT features are altered in older healthy participants as well as in ocular diseases, including slower dynamics, irregular oscillations, and reduced oscillation amplitude. The distinction between patients and healthy participants based on the calculation of the area under the curve of the receiver operating characteristics (AUC of ROC) were dependent on the pathologies and stimuli (0.7 < AUC < 1). PCT nevertheless provides relevant complementary information to assess the physiopathology of ocular diseases and to probe the functioning of retino-pupillary circuits.

## 1. Introduction

In the context of an increasing prevalence of ophthalmic diseases worldwide [1,2,3] designing fast, objective and convenient functional vision assessments are crucial for their detection, diagnosis and follow-up. Current functional vision assessments rely on subjective tests such as visual acuity, or standard automated perimetry (SAP) whose results can be hampered by fluctuations of attention, eye movements, fatigue, or stress. These tests are therefore not always reliable [4,5,6] nor are they sufficient to probe the visual health of individuals.

Recent advances in pupillometric research [7,8,9] and improvements of eye-tracking technologies open the way to novel functional vision assessments that can complement the existing ones. Fast, objective, pupillometry is gaining interest in ophthalmology, offering relevant tools to get insights into functional damage. The discovery of melanopsin within the body and arborescence of retinal ganglion cells that are intrinsically photoresponsive (ipRGCs) fostered a large number of studies aiming at uncovering their retinal connectivity and functioning as well as their functional roles [10,11,12,13,14,15,16]. Briefly, it was found that pupillary responses to light are mediated by specific melanopsin containing ganglion cells (ipRGCs) that project onto the Edinger-Whestphal nucleus through the PON which in turn projects on the ciliary ganglions that control the iris sphincters ipRGCs show slow intrinsic responses when stimulated by light in the blue range (melanopsin absorption peak ~480 nm), but are also extrinsically activated with faster time constants by stimulation with other wavelengths, through an indirect pathway involving rods, cones, and bipolar cells. As these different cells respond to different wavelengths, chromatic pupillometry developed in recent years, so as to probe these –intrinsic and extrinsic- circuits independently. As an example, stimulation with red light will indirectly stimulate ipRGCs through the activation of L-cones, while blue light stimulate ipRGCs [17,18].

**Figure 1:**
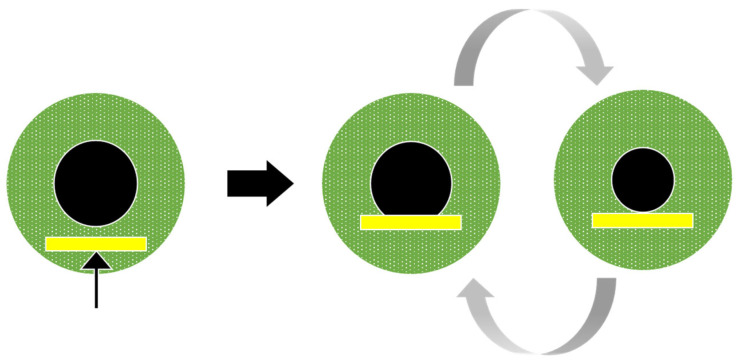
Pupil cycle oscillations elicited by positioning the beam of a slit lamp at the lower margin of the pupil. With this method, the pupil cycle time is derived from the number of cycles measured with a stop-watch over a fixed period of time. Note that quantification of amplitude and oscillations fluctuations is not available in this case.

We here present data characterising pupil cycle time (PCT) in several pathologies, using different spatial configurations and colours. Historically, periodic cycles of pupil dilation and constriction have been induced by illuminating the pupil margin with a thin beam from a slit lamp positioned so that pupil size regulates the amount of light entering the eye [19]: The light beam first induces pupil constriction, so that the light beam falls on the iris, outside the pupil, and does not stimulate the retina; the reduced retinal illumination in turn entail a pupil dilation, so that the light beam re-enters the eye, inducing constriction, and so on (Figure 1). This method was first used in optic neuritis, with the hypothesis that optic nerve demyelination in this disease would prolong PCT, which was indeed the case [20].

The measurement of PCT has been refined by several authors who used electronic devices to control the cycling. Subsequent investigations concerned a large event of neuropathies and neurological disorder, and used to probe the autonomous system. As a general rule, PCT was prolonged in Diabetic Retinopathy, Glaucoma, migraine, optic neuritis. Aging or exercise were also found to prolong the PCT [21,22,23,24,25,26,27,28,29].

Thus, PCT appears as a relevant functional test that evaluate the timing within a feedback loop involving i. information processing in the retina, ii. conduction time in the optic nerve and iii. dynamics within the pupillary circuits, including the Edinger-Westphal nucleus, ciliary ganglion, and iris sphincter. Damage related to a pathological condition known to target photoreceptors (e.g., AMD), ganglion cells (e.g., Glaucoma) or the optic nerve (e.g., optic neuritis, lateral sclerosis) is likely to perturb the processing within the retina and to alter the PCT. Furthermore, as the pupil controls the amount of light entering the eye so as to adapt to incoming illumination, an abnormal PCT may not only reveal a pathological condition but also characterise visual discomfort, glare or migraines, which are common complaints of patients whose origins are not always well understood and handled.

Despite its short duration (~1–5 min), the standard method of measuring PCT suffers from several limitations and issues:

- PCT measurements require good practice to master the slit lamp positioning, and any eye or head movement of the subject may disrupt the pupil cycling,
- The slit lamp stimulus is easily applied to the iris margin, but not so easily in other locations. As a result, the slit lamp stimulates only the peripheral retina and cannot be used to test specific retinal regions, or to investigate different stimulus characteristics (e.g., stimulus colour, size, structure, position or extent). With this method, the size of the retinal stimulus changes over time, so that a smaller retinal area is stimulated during constriction, possibly limiting the recruitment of the pupil arc.
- The outcome measure, pupil cycle time, is measured by counting the number of cycles over a fixed period of time using a stopwatch and dividing this number by the duration of the test. This method can only measure the cycle time—the period of the pupil oscillations- and does not allow quantitative assessment of other characteristics, such as pupil oscillation amplitude, regularity, mean pupil size or eye movements and number of blinks during the test.
- Studies using electronic devices to control pupil cycling often use a threshold to trigger the onset or offset of a beam light, resulting in square wave like stimulations.
- Different studies of PCT use different designs and displays. The characteristics of the slit lamp, such as their size or luminance, are not always reported, and the test duration varies. In addition, when the number of oscillations is counted by a human observer, small or irregular oscillations may be missed. Although it is often claimed that PCT could be used to test for a pathological condition, PCT measurements lack an agreed methodology that would be reliable and repeatable, so as to foster its routine use. As a result, there have been few publications on PCT since 2000, despite its recognised potential value.

### Computerized Induction of Pupillary Oscillations

**Figure 2:**
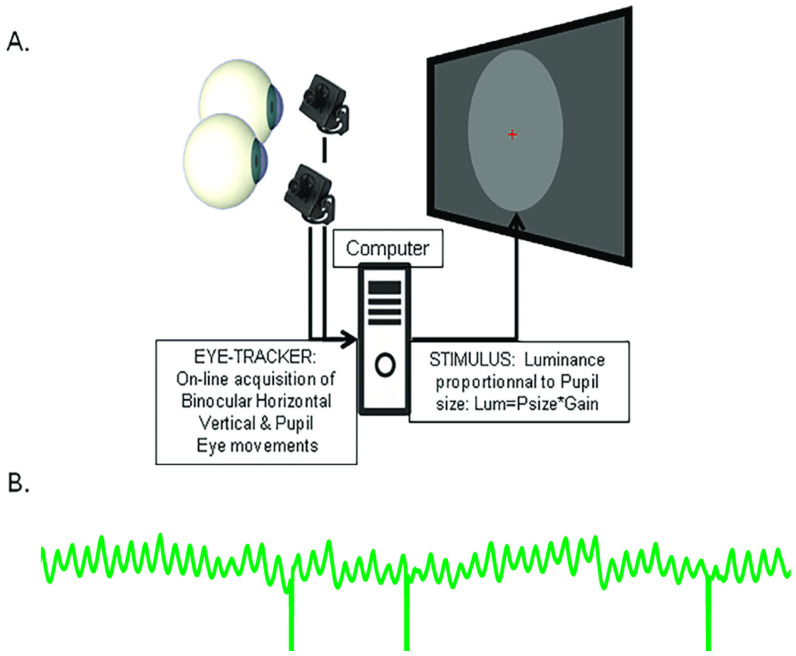
Illustration of the biofeedback setting used to induce pupil oscillations. (**A**) Pupil diameter measured by an eye tracker is converted in real time into a stimulus luminance proportional to pupil size, and updated on each frame. (**B**) Example of pupil oscillations: A large pupil results in a high stimulus luminance, which causes the pupil to constrict, resulting in a lower stimulus luminance which entrains a pupil dilation, and so on. Vertical lines are blinks.

Lamirel and colleagues [30] introduced a novel method that relies on computerised biofeedback, where online recording of pupil size with an eye tracker is used to adjust the luminance of a stimulus displayed on a computer screen in real time (Figure 2). In this setting, the luminance of a stimulus of any colour, size and position is set to be proportional to pupil size, and updated on every frame. With this closed loop coupling, a large pupil results in a high stimulus luminance, which in turn induces a pupil constriction, which in turn results in a lower stimulus luminance, and so on (see Supplementary Video S1). The so induced pupil oscillations that reflect the activation dynamics within the retino-pupillary circuits can be recorded, allowing quantitative off-line analyses to measure the frequency, amplitude and stability of pupil oscillations, as well as fixation eye movements and number of blinks, providing additional data relevant to the characterisation of eye health and potentially distinguishing patients from healthy individuals.

We used this method in three studies conducted with different participants on different sites. Study 1 involved young healthy participants (YHP) from the school of optometry of Université Paris-Saclay. The aim was to obtain baseline data and to characterise relevant pupil oscillation features. Study 2 was carried out at the Monticelli Clinic in Marseille on patients with Age-related Macular Degeneration (AMD) or Diabetic Retinopathy (DR), together with healthy participants (HP). Study 3 was conducted at the “Institut de la Vision” in Paris on three rare diseases: Retinitis Pigmentosa (RP), Stargardt Disease (SD), Leber Hereditary Optic Neuropathy (LHON) together with healthy participants (HP).

Depending on the study, stimuli of different sizes, configurations or colours were used both to deepen our investigations of PCT in different conditions and to test specific hypotheses. Our goal was to identify the stimuli best suited to elicit reliable oscillations in different pathological conditions, and to develop a convenient and fast method that can routinely be used in the clinic.

## 2. Material and Method

### 2.1. Study 1: Young Healthy Participants

Participants: A total of 36 students (age range 20–29, 18 females) in the school of optometry of Université Paris-Saclay with normal or corrected to normal vision were enrolled in this study. All underwent the PCT test, Optical Coherence Tomography (OCT) and ophthalmic examinations (visual acuity) as part of their university course. All participants provided informed written consent in accordance with the Declaration of Helsinki and were free to leave the study whenever they decided to do so. The study was approved by the local ethics committee “Comité de Protection des Personnes OUEST IV”, IRB #2020-A00859-30057.

Apparatus and Stimuli: Stimuli were displayed on a conventional monitor (Dell 2407WFPHC, 1024 × 768 × 8 bits refreshed at 60 Hz) placed at 57 cm from the eyes. Monocular eye movements and pupil size were recorded at 500 Hz with a Live Track Lightning remote eye tracker (Cambridge Research System Ltd., Rochester, UK, https://www.crsltd.com/tools-for-vision-science/eye-tracking/livetrack-lightning/, accessed 15 January 2025) placed 30 cm from the eyes. Recordings were down sampled to 60 Hz for the closed loop servo-coupling of stimulus luminance. A chin rest was used to stabilise participants’ eyes relative to the eye tracker. The stimulus was a grey disc (diameter ~35°, 700 pixels) presented at the centre of the screen. The PCT of each eye was recorded in two successive runs of 45 s.

### 2.2. Study 2: Age-Related Macular Degeneration and Diabetic Retinopathy

Participants: Participants were grouped as follows: AMD: 35 patients (mean age: 76 ± 6.3 years). DR: 34 patients (mean age: 60 ± 13.6 years). Healthy participants (HP): 42 subjects (mean age: 53 ± 14.5 years), meeting the following inclusion criteria: VA acuity greater or equal to 8/10, lack of previous ophthalmologic issue. The protocol conformed to the Declaration of Helsinki and was approved by a French Ethic Committee (“Comité de Protection des Personnes Ile-de-France III”, ID-RCB 2022-A00708-35). Participants were informed of the goal of the study and gave their written informed consent to perform the pupillary tests. All participants were free to leave the study whenever they decided to do so.

Apparatus and Stimuli: Hardware and settings were identical to those of Study 1, except that the display (Dell U2412, 1024 × 768, 60 Hz), eye tracker and chinrest were installed in an “Eye-Box” designed to isolate the participants and to limit the field of view. In addition, the stimuli were two rings (outer radius 33° inner radius 3.5°) with different colours (red and green) presented in succession for 20 s with a 4 s delay between runs. In each sequence, the luminance was modulated as a function of pupil size to elicit pupil oscillations. All tests were performed in a dim ambient light.

### 2.3. Study 3: Rare Pathologies: Retinitis Pigmentosa, Stargardt Disease and Leber Hereditary Optic Neuropathy

Participants: Fourteen patients with Retinitis Pigmentosa (RP: mean age: 41, sd: 11; 6 women), 14 patients with Stargardt disease (SD: mean age: 38, sd: 9; 5 women), 9 patients with Leber hereditary optic neuropathy (LHON: mean age: 33, sd: 7; 4 women) and 14 healthy participants (HP: mean age: 37, sd: 10; 6 women) were included in the study. All participants were aged between 20 and 58 years. The inclusion criteria for the healthy volunteers were a binocular corrected visual acuity greater or equal to 8/10 (≤0.1 logMAR) and a normal visual field (Supplementary Table S1). Patients and healthy participants were tested without corrections (lens or glasses) during the main experiment. The protocol conformed to the Declaration of Helsinki and was approved by the French local ethic committee (“Comité de Protection des Personnes Ile de France XI”, Ref CPP: 18002, Code: P1705, IDRCB: 2017-A03236-47; NCT04909398). All participants gave their written informed consent to participate in the study, and were free to leave the study whenever they decided to do so.

Apparatus and Stimuli: Stimuli were back-projected at 60 Hz onto a translucent screen (55.5 × 41.63 degrees visual angle) using a Titan 1080p video projector (Digital Projection, Ltd., Oldham, UK). Participants were seated comfortably at 128 cm from the screen with their head resting on a chin rest. Pupil modulations and eye movements of both eyes were recorded with an eye tracker (EyeLink II, 250 frames/s., SR Research, Ltd., Kanata, ON, Canada). The data were downsampled to 60 frames/s. The 2 cameras of the eye tracker were fixed to the chin rest and adjusted for each subject to obtain a good image of both pupils. Pupil oscillations were induced by monocular stimulation (right and left eye in succession). Each eye was stimulated independently, using a large movable black cardboard to mask the display screen to one eye.

Three different stimuli were used—a central disc, a peripheral ring and a full-field stimulus. The central disc covered 19° of visual angle. The inner and outer limits of the ring were 19° and 40° respectively. These stimuli could have different colours (blue, red, green, and grey) corresponding to modulations of the different RGB guns of the display. This design resulted in 12 conditions per eye, each lasting 45 s.

Features common to all studies: All experiments were controlled by custom software (Jeda [31]) running on Windows 10 (Microsoft Ltd., Redmond, WA, USA), which was used for stimulus generation and eye movement recording. A session started with positioning the participants and adjusting the eye tracker. After 5 to 10 min of adaptation to the low ambient light of the testing room, during which a questionnaire was given to the participants, a 5 points eye calibration procedure was performed. During the tests participants were asked to fixate the centre of the screen and to limit blinking as much as possible, with no other concurrent task.

Pupillary oscillations occur when the luminance of a stimulus is updated on each frame (at 60 Hz), as a function of the pupil size delivered by the eye tracker. These oscillation are produced because any pupil size fluctuation will entrain a change in luminance after a delay corresponding to the time required to convert luminance into retinal neural signals, propagation along the optic nerve, processing time within relay nuclei, and constriction or dilation of iris muscles. This so called Pupil cycle time thus reflects the functioning and integrity of the retino-pupillary circuits. The conversion from pupil size to stimulus luminance is achieved by multiplying the pupil size by a gain, G, chosen to keep the stimulus intensity in the range 1–255. Note that we directly modulated the RGB guns, not the luminance (in cd/m^2^ or any other unit). Therefore, the luminance changes were not linear. The reason for not using a gamma-corrected value is that a logarithmic compression is done within the retina [32].

The value of the gain, G, depends on the units of pupil size delivered by eye trackers. The gain was adjusted in preliminary experiments depending on the eye tracker used in each study and remained the same for all participants. The equation used for this conversion was of the form:

L_rgb_ = PS × G

with L corresponding to the stimulus intensity (value between 1 and 255 applied to the RGB guns), and PS is the size of the pupil delivered on-line by the eye-tracker. With the Live Track eye tracker (Studies 1 and 2), the square of the horizontal pupil diameter was first computed before applying the gain. With the EyeLink (Study 3), the gain was less than 1 as this eye tracker delivers the number of pixels detected by an algorithm as an estimate of pupil size (usually larger than 3000).

A brief rest between the different runs was used to change the stimulated eye (Right or Left in alternation).

The above summaries indicate that different stimuli were used in the different studies, performed at different times on different sites. This allowed to explore the effects of these different settings on PCT. As the studied diseases present different characteristics, we adapted the stimuli to test different hypotheses. For instance, in Study 3, we took into account both the spatial aspects of the damage described in LHON and SD (preponderant central damage) and thus used a central disc. Similarly, RP patients present damage to the peripheral retina, explaining why a peripheral ring was used to probe their PCT. The same logic applies to the choice of colours, as colour perception defects have been reported for some of the studied diseases [33,34,35,36,37,38,39].

### 2.4. Data Analyses

Data from the three studies were analysed in the same way using custom Matlab scripts (version R2018b, The MathWorks, Natick, MA, USA). Pupil oscillations were first extracted from the raw recordings. The traces were then corrected for blinks: A pre- and post-blink of 4 frames (66 ms) were used to isolate blinks. A smoothed linear interpolation was then applied to the blink sequences. We quantified the number data corrected for blinks as well as for eye movements. For the latter, we computed the standard deviation of horizontal and vertical eye positions and quantified saccades as horizontal and vertical eye positions more than 2 standard deviations from the mean eye position. We also characterised five Global Pupil State (GPS) variables measured on each stimulation sequence: slope of a linear fit of the pupil size, mean, maximum and minimum pupil size, and standard deviation of pupil size during oscillations.

The pupil oscillation maxima and minima were then determined for each corrected trace and used to calculate the oscillation period (mean of peak-to-peak duration), oscillation regularity (standard deviation of peak-to-peak duration), oscillation amplitude (mean of peak-to-trough amplitude) and amplitude variability (standard deviation of peak-to- trough amplitude). A Fourier transform was also computed on each trace and the maximum power and its corresponding frequency (in the range 0.2–2 Hz) were extracted. In addition, a mixed feature was computed corresponding to the product of power and frequency divided by the product of amplitude variability and period regularity:

MixVar = Pow × Freq/StdAmp × StdPeriod

This mixed variable is expected to characterise healthy participants who should have oscillations with a high power and a high frequency, both of which should be regular and stable over time (small amplitude standard deviation and small period standard deviation). Healthy participants are expected to have a high MixVar value whereas patients may present a lower MixVar value because power and frequency would be lower, and amplitude variability and period irregularity would be higher, as compared to healthy subjects.

In Study 1, one recording was removed from further analyses because of a technical issue. For studies 2 and 3, outliers were first detected and removed from each distribution of PCT features (data more than 3 scaled median absolute deviations). We checked the normality of each distribution with a Kolmogorov-Smirnov test. As all distributions were normally distributed, we used Student’s *t*-tests to compare each distribution of each variable from healthy participants (HP) with that of the patients. The Areas Under the Curve of Receiving Operating Characteristics (AUC of ROC) were computed (using the Matlab *figlm* and *percurve* functions) with varying subsets of PCT variables to determine whether these variables allow distinguishing healthy participants from patients. When possible, the functional characteristics of PCT were compared to the structural OCT data (RNFL) using Pearson’s correlation coefficients.

## 3. Results

**Figure 3:**
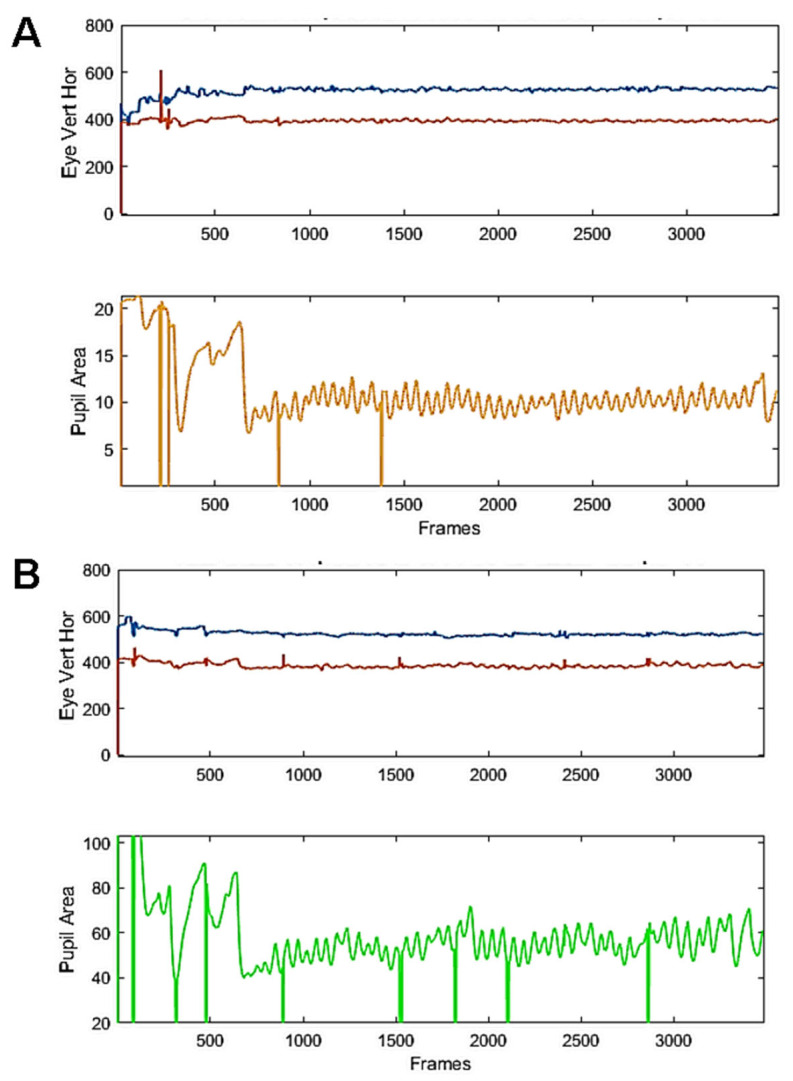
Examples of raw recordings from 2 young healthy participants. Eye movements (top, blue and red traces) and raw pupil traces (bottom, yellow or green) from 2 participants (**A**,**B**) during a run including a baseline fixation (3 s), the elicitation of a PLR (150 ms. flash, followed by 3 s of fixation) and the oscillatory phase, lasting 45 s. Vertical lines are blinks.

**Study 1:** In this study, the stimulus was a large grey disc presented in the centre of the display, whose luminance was set to be proportional to pupil size. Figure 3 present examples of raw traces from 2 participants recorded during a 45 s run. As it can be seen, regular, sustained and ample pupil oscillations were elicited with our setting. Blinks do not appear to notably disrupt the oscillatory behaviour.

**Figure 4:**
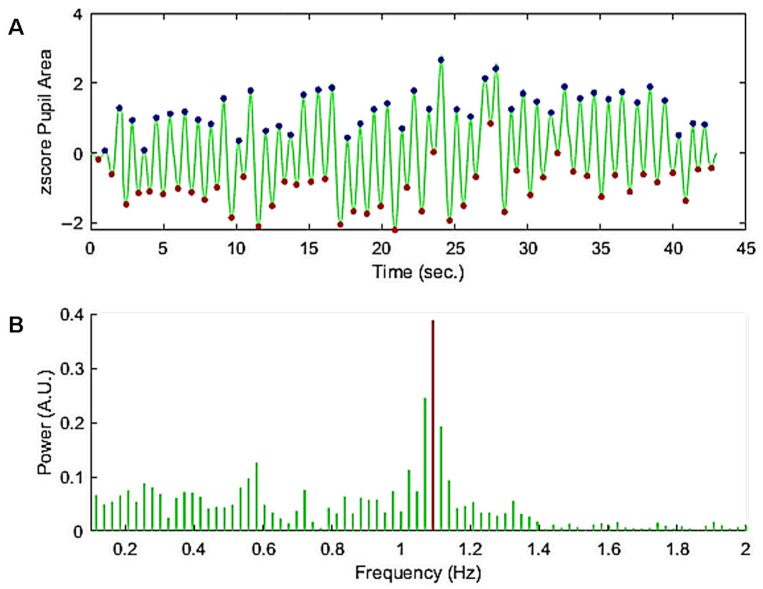
(**A**) Z-scored pupil oscillations corrected for blinks. A ramp from 0 to 1 and from 1 to 0 was applied to the beginning and end of the trace, respectively, to avoid spurious transients that could distort the spectral power distribution. The discs superimposed on the pupil trace show the maxima and minima of the oscillations (peak and trough times) used to compute the mean period and regularity over time, as well as the oscillation amplitude and variability. (**B**) Power spectrum between 0.2 and 2 Hz (green lines) of the trace in (**A**). The maximum power of the spectrum between 0.2 and 2 Hz (red line) was taken as a measure of the power and frequency of the pupil oscillation.

The raw recordings were segmented to isolate the onset of oscillations, the time when the feedback loop was enabled. Figure 4 shows an example of the z-scored corrected pupil signal with the maxima and minima of pupil oscillations (discs in Figure 4A) and the corresponding spectral power distribution (Figure 4B). Before computing the FFT a ramp from 0 to 1 and a ramp from 1 to 0 were convolved with the first and last second of the pupil trace to avoid spurious transients that could distort the spectral power distribution.

As it can be seen in this example, the peaks are regularly spaced in time despite changes in amplitude over time (Figure 4A). The Fourier spectrum of the corrected trace (Figure 4B) shows a clear peak in the spectral power distribution. The corresponding frequency bin was used to calculate the oscillation power and frequency.

**Figure 5:**
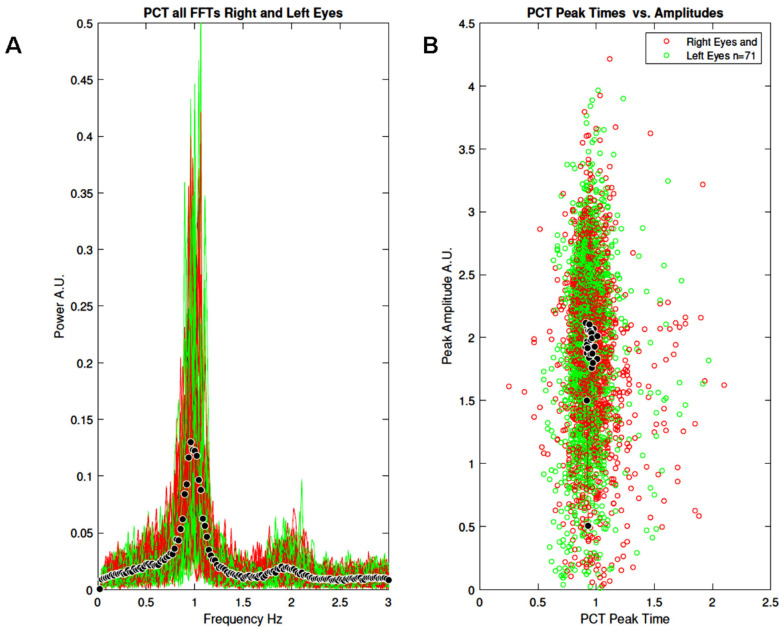
Results of study 1: (**A**) All FFTs normalised by frequency (power × frequency) for the right (red) and left (green) eyes (N = 71). Black symbols represent the mean FFT. (**B**) All peak times as a function of peak amplitudes for the right (red) and left (green) eyes (N = 71). Black symbols represent average peak time versus average peak amplitude.

Figure 5A shows all the FFTs from the right and left eyes (N = 71). Black symbols represent the average FFT. Figure 5B shows the distribution of peak times as a function of peak amplitudes. Black symbols represent average peak time vs. average peak amplitude. Although the spectral power shows large inter-individual variation, the oscillation frequency is very similar across participants, ranging from 0.9 to 1.2 Hz. Also note a peak of small amplitude in the 2 Hz range, suggesting the existence of power at the first harmonic. Similar observations can be made for the peak times (Figure 5B), all centred around 1 s, with the amplitude varying between participants.

**Figure 6:**
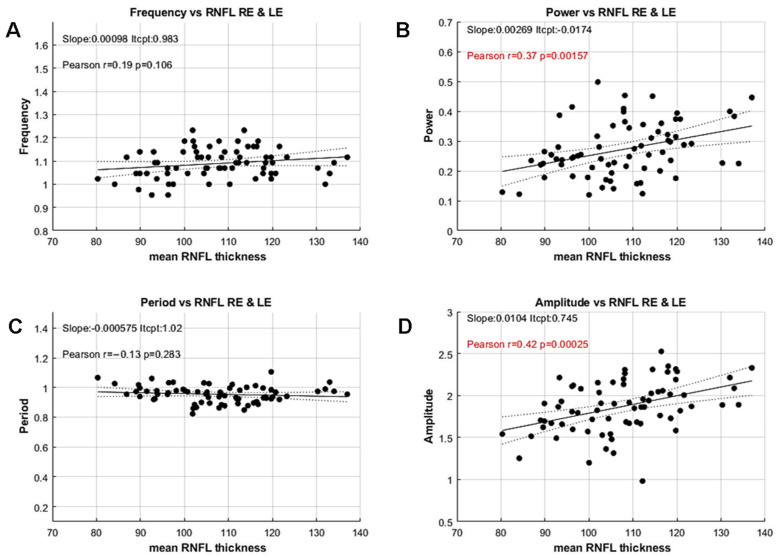
Correlations between the PCT variables and the mean RNFL thickness of both eyes. (**A**) Oscillation frequency versus mean RNFL. (**B**) Oscillation power versus mean RNFL. (**C**) Oscillation period versus mean RNFL. (**D**) Mean oscillation amplitude versus mean RNFL. Insets indicate the values of a linear fit, together with Pearson’s correlation coefficient, r. Significant values (*p* < 0.05) are shown in red. Filled and dashed lines show the linear fit to the data and the 95% confidence intervals, respectively.

We then compared these variables with the mean thickness of the Retinal Nerve Fiber Layer (RNFL) derived from the participants’ OCTs by computing Pearson’s correlation coefficients for each variable (Figure 6).

Pearson’s correlation coefficients between RNFL thickness and Frequency or Period, computed independently but reflecting a similar feature, are not significant (Frequency: r = −0.18; *p* = 0.48; Period: r = −0.1; *p* = 0.43). In contrast, the spectral power and the mean amplitude, computed with different methods but reflecting the same feature, are significantly correlated to the mean RNFL thickness (Power: r = 0.37, *p* < 0.002; Amplitude: r = 0.43; *p* < 0.001).

These results are noteworthy because the standard method for measuring PCT does not allow estimation of oscillation amplitude or power, and is therefore unable to uncover such an effect. Moreover, these correlations reveal a significant relationship between functional and structural data, even in this population of YHP, suggesting that PCT amplitude or power could be used as a proxy for RNFL thickness. The lack of correlation for period and frequency, together with the small inter-individual variability for these features, further suggests that the timing of the activity flow through retino-pupillary circuits is stable and independent of RNFL thickness in this population.

We observed, as expected, that the spectral power and mean amplitude, and the oscillation frequency and period, were highly correlated (r = 0.84; *p* < 0.0001; r = −0.8, *p* < 0.0001, respectively. Supplementary Figure S1). Therefore, in the following, we only consider the features derived from the oscillations peaks and troughs, including the mean amplitude and period and the amplitude variability and period regularity.

**Study 2:** This study aims to characterise PCT features in patients with AMD and DR, and to compare these data with adult healthy participants (HP). The stimuli were different from that of study 1, in that a green and a red discs were used in succession, instead of a grey disc. The reason for using coloured discs is that several studies have reported that chromatic vision can be altered in these pathologies. Using chromatic stimuli allows investigating whether PCT is differently altered in these diseases, as compared to HP. Moreover, as the Red and Green guns of the display deliver different luminance ranges, this design permits to determine the extent to which low or high luminance entail similar pupil oscillations. Although colour and luminance are confounding factors in this case, comparisons of patients with HP is still relevant.

**Figure 7:**
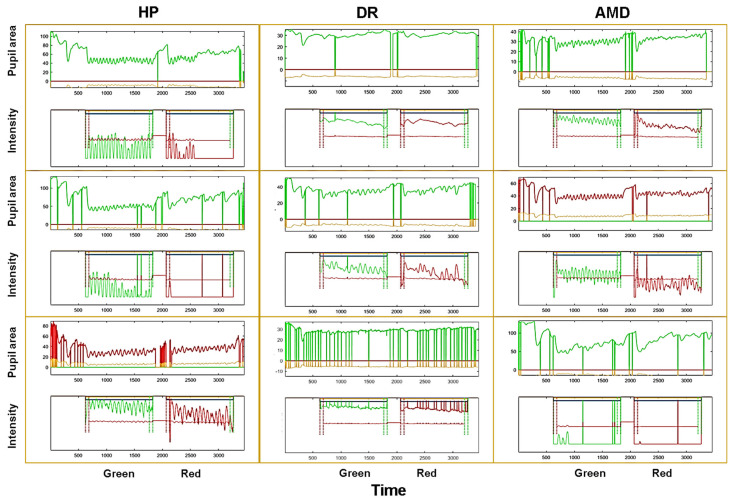
Examples of raw pupil recordings for 3 HP (left column), 3 AMD (middle column) and 3 DR (right column) patients. Each framed graph shows a complete trial, starting with the induction of a PLR, followed by the green stimulus, a 4 s delay, and the red stimulus. The upper trace is the pupil area, the lower trace is the corresponding RGB stimulus intensity modulation. Vertical lines are blinks.

Figure 7 shows examples of raw pupil oscillation traces for 3 healthy individuals, 3 AMD and 3 DR patients, illustrating the variability between and within each group. Overall, pupil oscillations were more difficult to entrain and were more irregular in this population as compared to YHP of study 1, and participants made more blinks. The red sequence hardly elicited oscillations in a number of participants, and the oscillations often fade away during the stimulation. These data were chosen to illustrate the existence of inter-individual variability not necessarily related to the subjects’ condition.

**Figure 8:**
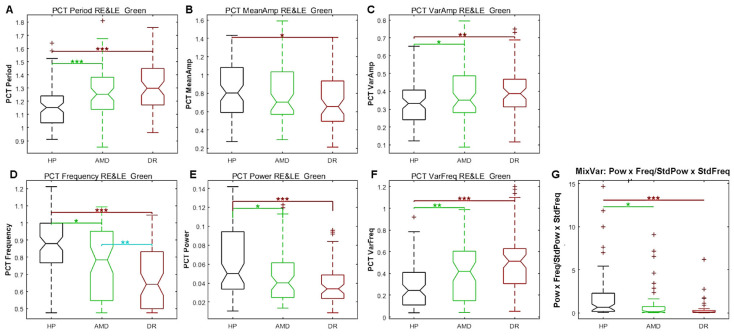
Study 2: Box plots of each PCT characteristics for the green sequence with pooled right and left eyes data. (**A**) Period. (**B**) amplitude. (**C**) Amplitude variability. (**D**) frequency. (**E**) Power. (**F**) Irregularity. (**G**) MixVar. Black plots: HP; Green plots: AMD; Red plots: DR. The statistical significance of comparisons between groups are shown by stars: * *p* < 0.05, ** *p* < 0.01, *** *p* < 0.001.

To compare the data from the different groups, we calculated Student’s *t*-test and Cohen’s *d* after removing outliers (data more than 3 median absolute deviations from the median) for each of the 7 distributions of PCT-relevant features. Some data sets could not be analysed because of too many blinks or technical issues (N = 3). Figure 8 shows the distributions of PCT variables for the green sequence and Table 1 summarises the statistical results for the red and green sequences.

As can be seen in Figure 8, the PCT for the green sequence is longer and the frequency is lower for patients than for HP. The mean PCT amplitude is different between HP and DR, but not between HP and AMD. The amplitude variability and period irregularity are less in HP than in patients. Consequently, the MixVar (see above) is greater in HP than in patients, as predicted.

Table 1 summarises significant differences between HP and AMD for the green and red sequence. AMD and HP differ in several respects for the green sequence but only the standard deviation of amplitude for the red sequence differed. For the HP and DR groups, significant differences and large effect sizes fare observed for the green sequence. DR also significantly differs from HP for several features of the red sequence with small effect sizes. Finally, the oscillation frequency significantly differs between DR and AMD, suggesting disease-dependent effects.

The results indicate that the PCT features for the red and green stimuli differentially elicit pupil oscillations depending on the participants’ condition. Most PCT variables for the green sequence differ significantly between HP and patients, with large effect sizes for some variables. This is not the case for the red sequence. The few significant effects for the latter may be explained by the difficulty of eliciting oscillations with a red stimulus regardless of the participants’ condition (see Figure 7), possibly because the gain used with this colour is not adapted (but see below the results of a control experiment), or because the red stimulus is less effective than the green stimulus in eliciting pupil responses. However, colour and luminance are confounding factors, so no firm conclusion can be drawn at this stage.

Pearson’s correlation coefficients between the PCT variables of the green and red sequence and RNFL values of AMD and DR patients were not significant (all *p* > 0.05). We could not perform OCT measures for HP, and thus could not evaluate correlations between PCT variables and RNFL for HP. The lack of significant correlations for AMD and DR contrast with the finding of significant correlations between PCT amplitude and RNFL in Study 1 for YHP. Several reasons could explain this difference, including the use of different stimuli, the variability of the patients’ condition or difference of age between these groups.

We further calculated AUCs of ROC to assess whether PCT variables could discriminate patients from HP. AUCs between HP and AMD, HP and DR and DR and AMD, were computed using all PCT features, for each eye separately. Despite the significant differences summarized above, AUCs of ROC were low (0.7 < AUC < 0.9) indicating that PCT features alone do not classify HP and patients with good sensitivity and specificity. However, when more variables are included (PCT variables plus Global Pupil State variables), AUCs of ROC for the right eye and green sequence is 0.94 for DR (sensitivity 0.88, specificity 0.94), and 0.91 for AMD (sensitivity 0.85, specificity 0.84). AUCs of 1 are found for AMD and DR when all variables and both eyes are included in the analyses. A summary of additional AUC of ROC analyses is available in Supplementary Table S2.

**Figure 9:**
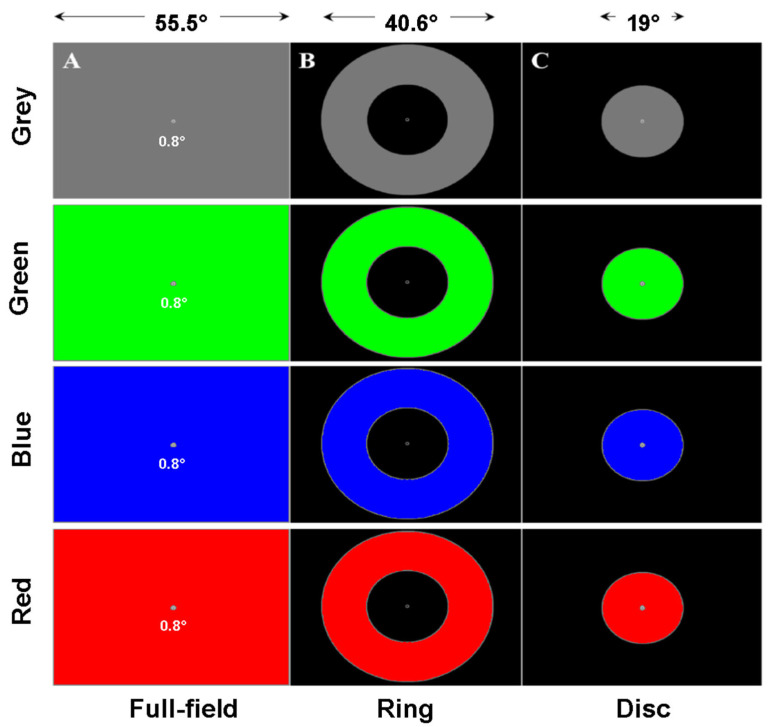
Illustration of the spatial configurations and colours used in Study 3. (**A**) Full-field stimulus of different colours. (**B**) Ring stimulus of different colours. (**C**) Disc stimulus of different colours.

**Study 3:** The third study used a larger set of spatial configurations and colours (Figure 9) to evaluate PCT in rare pathologies: Retinitis Pigmentosa (RP, N = 14, mean age: 41, sd: 11; 6 females); Stargardt disease (SD, N = 14, mean age: 38, sd: 9; 5 females) and Leber hereditary optic neuropathy (LHON, N = 9, mean age: 33, sd: 7; 4 females). Healthy matched participants were also included (HP, N = 14, mean age: 37, sd: 10; 6 females). All participants were aged between 20 and 58 years of age. Patients and healthy participants were tested without corrections (lenses or spectacles) during the main experiment.

RP is characterised by a loss of peripheral vision while central vision is spared. SD and LHON are associated with loss of central vision, with peripheral vision being less affected. Colour vision has also been found to be altered in these pathologies. In view of these observations (retinal distribution of the affected regions and colour vision deficits), we used several spatial configurations (full-field stimulus, FF, ring, PERI) and central disc C and colours: Red, Green, Blue or Grey. Each of the 12 conditions was tested for 45 s for each eye (24 trials per participant). Although the number of patients in each group is small, the fact that each participant ran 12 conditions per eye nevertheless allows conclusions to be drawn from pooled data (pooling eyes, configurations, colours, or the whole data set). Note however that the number of runs per condition per participant could vary, either because some participants were tired and did not run all conditions, or because eye data could not be recorded due to technical problems.

**Figure 10:**
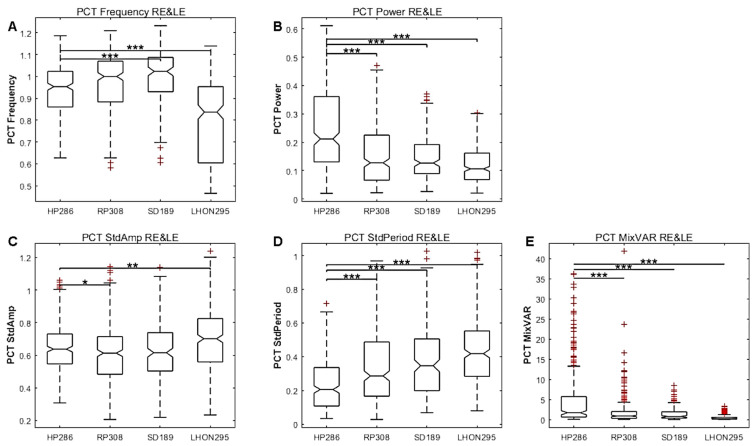
Study 3. Box plots of each population (HP, RP, SD, LHON) of PCT characteristics with data from the pooled right and left eyes. (**A**) Frequency. (**B**) Power. (**C**) Amplitude variability. (**D**) Regularity. (**E**) MixVar, equal to F × P/StdA × StdP. The statistical significance of comparisons between HP and patients are shown by stars: * *p* < 0.05, ** *p* < 0.01, *** *p* < 0.001.

Figure 10 shows the results for the pooled PCT data (configurations, colours and eyes), together with significant differences between HP and patients groups. As it can be seen, most variables show significant differences between HP and patients, including frequency, power and amplitude, variability and regularity, as well as the MixVar variable.

Table 2 summarises the statistical comparisons between HP and patients performed after pooling the data from all conditions and eyes. Table 3 shows similar statistics for the different spatial configurations and Table 4 the results for different colours. Note that the number of observations may differ in each table and across conditions, as not all participants ran all conditions and because some trials were excluded from the analyses due to technical problems with the eye tracking recording or a very large number of blinks.

The distributions of PCT variables as a function of spatial configurations or colours for each pathology are available in Supplementary Figures S2 and S3, and in a previous description of this study.

Table 2, Table 3 and Table 4 show that several PCT features are significantly different for each patients group as compared to HP, although with disease and configuration dependent effects. For the RP group, most PCT variables differ from HP, with large effect sizes on average. This observation holds for all configurations and for all colours. For SD and LHON, the period and frequency do not significantly differ from HP, but differ significantly for several other variables.

Considering the statistics for the whole data set of Table 2 indicates that the amplitude, the variability and the regularity (estimated as the standard deviation of peak-to-peak time intervals) are less for all patients as compared to HP, while the period differs only for RP patients. The MixVar is significantly different from HP for the LHON and RP groups. These conclusions are slightly different for the different spatial layout (Table 3) and different colours (Table 4), but the amplitude and oscillation regularity differ for almost all conditions and diseases. Note the significant effects with large effect sizes for the period of the red stimuli (Table 4), suggesting that red stimuli elicit irregular, slower and smaller oscillations, in line with the conclusion of Study 2 (also see Supplementary Figures S2 and S3).

AUCs of ROC were computed in different ways with this data set. We first tested PCT variables only, using data from all configurations and colours with all the patients (N = 995) relative to HP (N = 341). AUC of ROC was 0.75 (sensitivity 0.97 specificity 0.23). AUC of ROC using additional variables (GPS and NCOR) grew to 0.81 (CI: 0.78–0.84, sensitivity 0.96 specificity 0.32). AUCs of ROC computed separately for the different diseases and/or specific stimuli (spatial configurations, or colours) give better results, and reflect to some extent the specificity of each disease. For instance, for RP with poor peripheral vision but preserved central vision, using only the data from the RING stimulus gives an AUC of 0.94 (CI_95_ 0.86 and 0.97; sensitivity: 0.93; specificity: 0.81), but the AUC is only 0.88 (CI_95_ 0.82 and 0.92; sensitivity: 0.80; specificity: 0.84) for the central disc.

### Control Analyses Common to All Studies

We wondered whether blinking frequency—and the associated data correction procedure- could alter the characteristics of PCT oscillations. To address this question, we analysed the relationships between the percentage of data corrected for blinks and PCT amplitude, frequency and power. We did so for study 2, pooling all the available data in each case. No effect of the number of corrected data on PCT was found (Supplementary Figure S4).

Whether the gain used to transform pupil size into luminance influences PCT measures was addressed in a control experiment (see Supplementary Materials). As expected, different gains changed the mean stimulus luminance, but sustained oscillations were elicited for all gains greater than 1 and less than 10 (Supplementary Figures S5 and S6), indicating that the choice of gain is unlikely to account for the present results. In particular, different gains are unlikely to account for the increased amplitude variability and decreased oscillation regularity seen in diseases as compared to HP. In Studies 2–3, oscillation regularity and amplitude stability are significantly better in HP as compared to diseases (see Table 1, Table 2, Table 3 and Table 4) and are therefore PCT features of interest that are distinctive characteristics of diseases.

## 4. Discussion

Real-time biofeedback coupling pupil size to stimulus luminance evoked pupil oscillations around 1 Hz under different stimulus conditions, including different sizes, colours, and layout configurations. Study 1 shows that YHP exhibit highly regular oscillations over time, with small inter-individual differences. Pupil power and oscillation amplitude are more variable, and this variability is significantly correlated with RNFL thickness in this population (Study 1). Studies 2 and 3 shows that patients’ pupil oscillations differ from those of HP in several respects, including variables not accessible in previous studies using the standard methods described in the introduction (Oscillation regularity and amplitude variability for instance). These results suggests that the flow of activity within retino-pupillary circuits is perturbed in the 5 pathological conditions studied, and that assessment of PCT provides relevant functional information related to the physiopathology of these diseases. Crucially, PCT power and amplitude, as well as PCT regularity and variability are most affected in diseases, while PCT frequency and periodicity are not always at stake, suggesting that timing variables remain comparatively less perturbed. Red stimuli are less efficient at inducing oscillations than other colours, and this effect is more pronounced in patients. This finding is unlikely to solely result from a lower luminance of red stimuli as compared to high green luminance, as a blue low luminance stimulus elicits overall more reliable oscillations. We speculate that this difference could be due to both a weaker contribution of L-cones to pupillary responses and to augmented disease related damage of these photoreceptors.

Altogether, we provide a comprehensive data set showing the selective distributions of deficits related to the particular spatial and chromatic contributions to endogenous pupil oscillations. For instance, DR patients of Study 2 show overall poor pupil reactivity, consistent with previous studies. Our results with AMD, RP, SD and LHON are overall consistent with other pupillometric findings [40,41,42,43,44,45,46,47,48,49,50].

These different results underline the interest of our method which provides quantitative estimates of features that are not accessible with the standard PCT induction methods. However, the averaged PCT in Study 1, about 1000 ms, is somewhat longer than the PCT values reported in other studies, in the order of 700–950 ms,. Several reasons can account for this difference. The age of the participants, known to slow down the PCT, and the method and stimuli were different. When measured with a slit lamp positioned at the iris margin, the size of the stimulus on the retina changes with oscillations, not its luminance. In addition, past studies do not always report the size and luminance of the slit lamp that may differ between studies. The number of cycles during a fixed time window is counted by the operator, so that cycles with a small amplitude or an irregular period may be missed. Finally, this method involves peripheral retinal illumination and not a stimulation of the macula, which is known to respond more strongly than the peripheral retina [51,52,53]. Other studies have used an electronic device to control a LED, using a threshold to trigger the onset and offset of the LED. There are several issues with the described methods, such as the LED colour or delay needed to control the LED (red LED, 140 ms delay and ON/OFF luminance changes), or the protocol design. These differences are noteworthy, and may explain the diffrences between previous and the present results.

## 5. Limitations

The fact that we conducted studies on different sites with different displays using different stimuli limits the ability to reliably compare the results of the different populations. In addition, the ophthalmic examinations were different and sometimes incomplete (e.g., no OCT data for HP in Study 2, lack of visual field examination). Thus, comparing the functional pupil data and other functional and structural data is limited. The use of RGB gun modulations, and of colours whose chromatic spectra are not well characterised and have different luminance also limit the conclusions that can be drawn from Study 3 about the effects of chromatic content.

An unknown but interesting issue that, to our knowledge, has not been addressed is the effect that cognitive functions—attention, memory, cognitive load—might have on PCT oscillations.

Finally, PCT reflects the overall timing and function of several different components recruited sequentially: retinal processing, retinal and optic nerve conduction delays, relay nucleus processing (PON, Edinger-Whestphal, ciliary ganglion), and iris muscle efficacy and time constant. PCT does not allow the discrimination of these components to identify the underlying physiopathology without additional work necessary to better isolate the retinal contributions of rods, cones or ganglion cells (including ipRGCs) to the PCT measures. However, PCT is quick and easy to use and contributes to the functional characterisation of pathological conditions in a simple and rapid manner that does not rely on subjective responses.

## 6. Conclusions

Pupil Cycle Time, as measured here, provide relevant functional measures that characterise the integrity of retinopupillary circuits in healthy participants and in patients with retinopathies or neuropathies. Although the biofeedback setting requires an eye tracker, it provides a wealth of data not available with standard functional (Visual acuity, perimetry) or structural methods (OCT). PCT variables correlate to some extent with structural features measured with OCT, at least in young healthy participants. The capability of PCT variables to distinguish healthy participants from patients with an ophthalmic condition (0.7 < AUC < 0.9) indicates good performance depending on the variables included in the analyses. Although the AUC values reported here are sometimes modest, PCT variables brings additional information on the condition of patients relative to HP: it does inform on the functioning of the retino-pupillary circuits, which is relevant in itself and rarely evaluated per se. As a matter of fact, malfunctioning retino-pupillary circuits can contribute to visual discomfort or glare. PCT could also be measured in a variety of ophthalmic or neurologic diseases, as well as in subject complaining from migraine or photophobia. The method presented here is fast, objective, non-invasive and requires little expertise, opening the way to its routine use in clinical practice.

## Figures and Tables

**Table 1:** Summary statistics for the Green and Red stimulation sequences for the pooled left and right eyes. Comparisons of data for HP (83 eyes) versus AMD (68 eyes), HP versus DR (68 eyes), and AMD versus DR. The left column shows the PCT characteristics of interest. Significant differences (*p* < 0.05) are shown in green, and Cohen’s *d* above or below 0.6 are shown in yellow.

**PCT Variables RE&LE Green**	**HP n83 vs. AMD n68**		**HP n83 vs. RD n68**		**RD vs. AMD**	
	***p***	**Cohen’s *d***	***p***	**Cohen’s *d***	***p***	**Cohen’s *d***
**Period**	0.000	−0.610	0.000	−0.904	0.132	−0.264
**StdPeriod**	0.001	−0.554	0.000	−0.916	0.058	−0.329
**Amplitude**	0.493	0.113	0.016	0.399	0.119	0.272
**VStd Amplitude**	0.019	−0.395	0.003	−0.514	0.656	−0.079
**MixVar**	0.034	0.425	0.005	0.534	0.573	0.108
**PCT Variables RE&LE Red**	***p***	**Cohen’s *d***	***p***	**Cohen’s *d***	***p***	**Cohen’s *d***
**Period**	0.730	0.057	0.214	−0.210	0.131	−0.266
**StdPeriod**	0.923	−0.016	0.004	−0.495	0.006	−0.485
**Amplitude**	0.029	0.361	0.025	0.371	0.978	0.005
**VStd Amplitude**	0.165	0.231	0.761	−0.050	0.146	−0.253
**MixVar**	0.407	0.156	0.818	0.041	0.536	−0.117

**Table 2:** Summary statistics computed on the whole data set of Study 3. The leftmost column indicates the PCT features of interest. Column titles indicate the comparisons done with the number of runs for each group. The probability of a significant difference (Student’s *t*-test, *p* values < 0.05 in green), and the effect size (Cohen’s *d*, values greater than 0.6 of lower than −0.6 in yellow) for each variable.

	HP n 297 vs. SD n 331		HP n 297 vs. LHON n 202		HP n 297 vs. RP n 321	
PCT Variables RE&LE ALL	*p*	Cohen’s *d*	*p*	Cohen’s *d*	*p*	Cohen’s *d*
**Period**	0.099	−0.13	0.724	0.03	0.000	−0.894
**Std Period**	0.000	−0.49	0.000	−0.67	0.000	−1.08
**Mean Amplitude**	0.000	0.69	0.000	0.79	0.000	1.22
**Std Amplitude**	0.014	0.20	0.808	0.02	0.002	−0.249
**MixVar**	0.309	0.082	0.046	0.184	0.000	0.45

**Table 3:** Same as Table 2 for the different spatial configuration: Full-field, Ring, Central disc. The probability of a significant difference (Student’s *t*-test, *p* values < 0.05 in green), and the effect size (Cohen’s *d*, values greater than 0.6 of lower than −0.6 in yellow) for each variable.

**Full-Field**	**HP n108 vs. SD n118**		**HP n108 vs. LHON n70**		**HP n108 vs. RP n120**	
**PCT Variables RE&LE**	***p***	**Cohen’s *d***	***p***	**Cohen’s *d***	***p***	**Cohen’s *d***
**Period**	0.171	0.19	0.003	0.47	0.000	−0.64
**Std Period**	0.019	−0.32	0.000	−0.60	0.000	−1.18
**Amplitude**	0.000	0.71	0.000	0.82	0.000	1.53
**Std Amplitude**	0.055	0.26	0.318	−0.15	0.026	−0.30
**MixVar**	0.616	0.07	0.064	0.29	0.000	0.53
**Ring**	**HP n93 vs. SD n106**		**HP n93 vs. LHON n64**		**HP n93 vs. RP n101**	
**PCT Variables RE&LE**	***p***	**Cohen’s *d***	***p***	**Cohen’s d**	***p***	**Cohen’s *d***
**Period**	0.170	−0.20	0.252	−0.19	0.000	−1.62
**Std Period**	0.000	−0.56	0.000	−0.86	0.000	−1.55
**Amplitude**	0.000	0.61	0.000	0.86	0.000	1.28
**Std Amplitude**	0.901	−0.02	0.498	−0.11	0.081	−0.26
**MixVar**	0.398	0.12	0.202	0.213	0.003	0.44
**Disc**	**HP n85 vs. SD n94**		**HP n85 vs. LHON n58**		**HP n93 vs. RP n86**	
**PCT Variables RE&LE**	***p***	**Cohen’s *d***	***p***	**Cohen’s *d***	***p***	**Cohen’s *d***
**Period**	0.004	−0.44	0.097	−0.29	0.000	−0.61
**Std Period**	0.000	−0.71	0.000	−0.71	0.000	−0.64
**Amplitude**	0.000	0.87	0.000	0.90	0.000	0.90
**Std Amplitude**	0.139	0.22	0.039	0.36	0.120	−0.24
**MixVar**	0.276	0.16	0.990	0.00	0.031	0.33

**Table 4:** Same as Table 2 for the different hues: Blue, Red, Green and Grey runs, each indicated by the top colored line. The probability of a significant difference (Student’s *t*-test, *p* values < 0.05 in green), and the effect size (Cohen’s *d*, values greater than 0.6 of lower than −0.6 in yellow) for each variable.

**PCT Variables RE&LE**	**HP n63 vs. SD n66**		**HP n63 vs. LHON n38**		**HP n 63 vs. RP n57**	
**Blue**	***p***	**Cohen’s *d***	***p***	**Cohen’s *d***	***p***	**Cohen’s *d***
**Period**	0.283	−0.19	0.116	−0.33	0.000	−1.07
**Std Period**	0.013	−0.45	0.000	−0.78	0.000	−1.03
**Amplitude**	0.000	0.79	0.000	1.12	0.000	1.11
**Std Amplitude**	0.145	0.26	0.702	−0.08	0.002	−0.58
**MixVar**	0.584	0.10	0.561	0.12	0.002	0.57
**Red**	**HP n61 vs. SD n60**		**HP n61 vs. LHON n37**		**HP n61 vs. RP n45**	
	***p***	**Cohen’s *d***	***p***	**Cohen’s *d***	***p***	**Cohen’s *d***
**Period**	0.000	−0.73	0.262	−0.24	0.003	−0.61
**Std Period**	0.000	−1.14	0.000	−0.83	0.000	−0.89
**Amplitude**	0.000	1.08	0.000	1.06	0.000	1.14
**Std Amplitude**	0.691	−0.07	0.131	0.32	0.118	0.31
**MixVar**	0.916	−0.02	0.911	0.02	0.279	0.21
**Green**	**HP n99 vs. SD n126**		**HP n99 vs. LHON n71**		**HP n99 vs. RP n126**	
	***p***	**Cohen’s *d***	***p***	**Cohen’s *d***	***p***	**Cohen’s *d***
**Period**	0.355	−0.13	0.967	0.01	0.000	−0.97
**Std Period**	0.004	−0.40	0.000	−0.58	0.000	−0.89
**Amplitude**	0.000	0.49	0.003	0.48	0.000	0.84
**Std Amplitude**	0.002	0.41	0.095	0.26	0.048	−0.27
**MixVar**	0.826	−0.03	0.051	−0.31	0.034	0.29
**Gray**	**HP n97 vs. SD n108**		**HP n97 vs. LHON n68**		**HP n97 vs. RP n120**	
	***p***	**Cohen’s *d***	***p***	**Cohen’s *d***	***p***	**Cohen’s *d***
**Period**	0.496	0.10	0.268	0.18	0.000	−0.97
**Std Period**	0.035	−0.30	0.000	−0.64	0.000	−1.21
**Amplitude**	0.000	0.60	0.000	0.90	0.000	1.54
**Std Amplitude**	0.277	0.15	0.920	−0.02	0.005	−0.39

## Data Availability

The data are available from the corresponding author on reasonable request.

## Supplementary Materials

The following supporting information can be downloaded at: https://www.mdpi.com/article/10.3390/vision9030051/s1, Video S1: Example of binocular pupil oscillations; Table S1: Demographics of the participants of Study 3; Figure S1: Correlation between PCT spectral power and amplitude; Figure S2: Distributions of PCT values for different spatial configurations and each group of Study 3; Figure S3: Distributions of PCT values for different colours and each group of Study 3; Figure S4: PCT variables as a function of the number of corrected data in Study 2; Figure S5: Raw traces of pupil oscillations with different gains; Figure S6: Distribution of PCT variables as a function of gain.
