# Supplementary material for: Uncovering the Characteristics of Pupil Cycle Time (PCT) in Neuropathies and Retinopathies
Source: Vision (Basel). 2025 Jun 30;9(3):51. doi: 10.3390/vision9030051 (PMC12286252; doi:10.3390/vision9030051)
Supplement: Supplementary file 1 [file vision-09-00051-s001.zip › PCT newSupdataLorenceau2025 - edit.pdf]

Uncovering the characteristics of Pupil Cycle Time (PCT) in neuropathies and retinopathies

Laure Trinquet[8], Suzon Ajasse[2], Frédéric Chavane[3], Richard Legras[4], Frédéric Matonti[5], José-Alain Sahel[6], Catherine Vignal-Clermont[7] and Jean Lorenceau[1*]

Supplementary Materials

Supplementary Movie #1: Example of binocular pupil oscillations induced with a biofeedback setting where pupil size measured with an eye tracker is converted into stimulus luminance in real time. The movie shows the recorded eye (yellow circle) and the fellow eye.

Supplementary Tables

General				Refraction RE			Refraction LE			Visual Acuity (LogMAR)			Pelli-Robson (logCS)			Summary	
n°	Sex	Age	Pathology	Sphere	CYL	Axis	Sphere	CYL	Axis	Right Eye	Left Eye	Binocular	Right Eye	Left Eye	Binocular	Age	Sex
22	M	30	HP	NA	NA	NA	NA	NA	NA	-0,3	-0,2	-0,3	1,65	1,65	1,65	37 +-10	8M / 6F
25	M	27	HP	NA	NA	NA	NA	NA	NA	-0,2	-0,2	-0,24	1,95	1,95	1,95	Max 58	
29	M	58	HP	-2,5	0	0	-2,25	-0,5	125	0	-0,08	-0,1	1,95	1,95	1,95	Min 24	
30	M	50	HP	0	0	0	0	0	0	-0,1	-0,1	-0,2	1,95	1,95	1,95		
35	F	42	HP	0	0	0	0	0	0	-0,02	-0,1	-0,12	1,95	1,95	1,95		
36	M	40	HP	-2	-0,5	84	-1,5	-0,5	100	-0,16	-0,16	-0,2	1,95	1,95	1,95		
37	F	37	HP	-10,25	-1,25	157	-9,5	-1,75	172	0	-0,04	-0,1	1,95	1,95	1,95		
38	F	38	HP	0	0	0	0	0	0	-0,16	-0,12	-0,18	1,95	1,95	1,95		
39	F	45	HP	0	0	0	0	0	0	0	0,04	-0,04	1,95	1,95	1,95		
40	M	35	HP	0	0	0	0	0	0	-0,06	-0,1	-0,2	1,95	1,95	1,95		
44	M	34	HP	0	0	0	0	0	0	-0,16	-0,2	-0,26	1,95	1,95	1,95		
46	F	34	HP	-5,5	-0,5	166	-5	-1,5	43	0	-0,1	-0,1	1,95	1,95	1,95		
54	F	24	HP	0,5	-0,25	106	0,75	0	0	-0,2	-0,12	-0,26	1,95	1,95	1,95		
55	M	24	HP	0	0	0	0	0	0	-0,3	-0,3	-0,3	1,95	1,95	1,95		
3	M	42	SD	+0,5	0,25	80	+0,5	0,25	175	0,86	0,76	0,78	1,65	1,65	1,65	38+-9	10M / 4F
4	M	42	SD	-0,25	0,25	65	-0,75	0,25	5	0,3	0,4	0,2	1,5	1,35	1,65	Max 55	
12	F	33	SD	-2	-0,5	75	-2,25	-1	90	1,02	1,02	1	1,2	1,2	1,2	Min 21	
15	F	46	SD	-1,25	-0,5	80	-1,75	-0,75	25	0,86	0,74	0,74	1,5	1,65	1,5		
27	M	31	SD	-1,75	-0,75	20	-2	-0,5	160	0,9	0,9	0,9	1,35	1,35	1,35		
28	M	45	SD	1,75	-1,5	25	2,5	-2,25	170	1	1,1	1,1	1,35	1,35	1,35		
31	M	21	SD	-2,5	-1,75	170	-1,5	-2	175	0,9	0,9	0,9	1,5	1,65	1,65		
32	M	21	SD	-1,5	-1,75	180	-1,25	-3	180	0,94	0,96	0,86	1,5	1,2	1,5		
41	F	33	SD	4	-2	180	5	-2,25	5	1,04	1,18	1	1,5	0,9	1,35		
42	M	37	SD	5,5	-1,75	15	7	-0,5	7	1,26	1,16	-	0,75	0,6	DM		
43	M	35	SD	0	-1,5	170	-1,5	-0,5	170	1,04	0,92	0,92	1,05	1,2	1,35		
50	M	49	SD	-3	-0,75	105	-3,5	-0,25	140	1	1,16	1,02	1,35	1,2	1,35		
51	F	42	SD	0,5	-1,25	25	0,75	-1,25	150	1	1	1	1,05	1,05	1,2		
53	M	55	SD	0,75	-0,75	90	0,75	-0,5	115	1,1	1,3	1,2	1,2	1,35	1,35		
2	F	24	RP	-1,75	2	5	-1,75	2,5	165	0,26	0,34	0,22	0,9	0,9	1,2	41+-10	8M / 6 F
6	M	35	RP	-7,25	-2,25	20	-8	-2	175	0,38	0,42	0,42	1,65	1,35	1,65	Max 58	11
7	M	37	RP	-4,75	3,25	130	-6,25	2,5	25	0,72	0,76	0,66	0,45	0,15	0,45	Min 24	
8	M	41	RP	-5,5	-0,25	160	-4	-1,25	20	0,04	0,04	0,02	1,65	1,65	1,65		
9	M	29	RP	0	0,5	100	-0,5	0,5	110	-0,04	-0,08	-0,08	1,65	1,65	1,65		
10	M	53	RP	-6	1,5	10	-3,5	0,75	180	0,84	1,02	0,78	0,45	0,3	0,45		
11	M	50	RP	-0,5	1	125	-2	1,25	10	0,72	0,7	0,68	0,45	0,6	0,6		
13	F	58	RP	-2,75	-1,5	85	-3,25	-1,5	80	0,06	0,22	0	1,65	1,65	1,65		
14	M	56	RP	-1,25	-1,75	90	0,75	-0,5	115	0,28	-0,04	-0,04	1,65	1,65	1,65		
16	F	53	RP	-0,75	-1,75	75	-0,75	-2	105	0,34	0,22	0,3	1,65	1,5	1,65		
17	F	38	RP	-6	-2,5	20	-6,5	-2,25	155	0,34	0,44	0,34	1,35	1,05	1,35		
18	F	24	RP	-6	-2,25	15	-6	-1	135	0,22	0,12	0,1	1,65	1,5	1,65		
19	M	36	RP	-3,5	-1	0	-2,25	-1,25	5	0,36	0,12	0,1	1,5	1,65	1,65		
24	F	39	RP	NA	NA	NA	NA	NA	NA	0,3	0,4	0,3	1,65	1,65	1,65		
1	M	36	LHON	-0,5	0,75	80	-0,25	1,75	90	0	0,1	0	1,05	1,05	1,05	33 +-7	5M / 4F
20	F	28	LHON	-1,5	-0,25	15	-1	-0,25	15	0,32	-	0,32	0,45	DM	0,45	Max 42	
23	M	33	LHON	1,25	NA	NA	1,25	NA	NA	1,52	1,48	1,48	0	0,45	0,45	Min 20	
26	F	40	LHON	2,5	0	0	3,25	-0,5	135	1,4	1,3	1,4	0,75	1,05	0,75		
45	F	42	LHON	-0,75	-0,25	130	-0,75	-0,5	15	1,02	1,1	0,92	1,05	1,05	1,35		
47	M	34	LHON	-3	-1,25	5	-2,75	-0,25	125	1,44	1,32	1,36	0,6	0,45	0,75		
48	M	20	LHON	0,25	0	0	0	0	0	1,04	0,24	0,44	0,15	0,15	0,15		
49	F	33	LHON	-0,5	-0,75	100	-1	-0,5	75	1,5	1,26	1,32	0,15	0,6	0,45		
52	M	31	LHON	0	-0,75	10	0,25	-1	10	1	0,96	0,96	1,35	1,5	1,65		

HP : Healthy participants
SD : Stargardt disease
RP: Retinitis Pigmentosa
LHON : Leber Heriditary Optic Neuropathy

Supplementary Table 1: Demographics of the participants of Study 3

Filter for AUC of ROC GREEN	Diabetic Retinopathy vs HP						Age-related macular degeneration (AMD) vs HP					
	AUC	CI95% low	CI95% high	Sens	Spec	EffSize	AUC	CI95% low	CI95% high	Sens	Spec	EffSize
**RE** PCT	0.92	0.8	0.97	0.85	0.87	0.964	0.84	0.74	0.91	0.61	0.95	0.69
**RE** PCT, GPS	0.94	0.79	0.98	0.88	0.94	0.87	0.91	0.87	0.98	0.85	0.84	0.876
**RE** PCT, GPS, COR	**1.00**	**1.00**	**1.00**	**1.00**	**1.00**	**1.71**	**1.00**	**1.00**	**1.00**	**1.00**	**1**	**1.2**
**LE** PCT	0.85	0.73	0.93	0.67	0.93	0.56	0.73	0.60	0.88	0.74	0.69	0.255
**LE** PCT, GPS	0.88	0.72	0.95	0.68	1.00	0.52	0.89	0.80	0.95	0.70	0.92	0.291
**LE** PCT, GPS, COR	**1.00**	**1.00**	**1.00**	**1.00**	**1.00**	**1.25**	**1.00**	**1.00**	**1.00**	**1.00**	**1**	**1.08**
**RE&LE** PCT	0.86	0.79	0.92	0.65	0.94	0.73	0.83	0.78	0.91	0.68	0.82	0.446
**RE&LE** PCT, GPS	0.91	0.86	0.94	0.77	0.89	0.71	0.87	0.79	0.94	0.84	0.81	0.86
**RE&LE** PCT, GPS, COR	**0.97**	**0.92**	**0.99**	**0.90**	**0.96**	**1.44**	**1.00**	**1.00**	**1.00**	**1.00**	**1**	**0.97**
**RE&LE** GPS, COR	0.95	0.88	0.98	0.85	0.97	0.78	0.90	0.84	0.85	0.81	0.84	1.04
**DR** vs **AMD** **RE&LE** PCT , GPS, COR	0.95	0.88	0.98	0.85	0.97	0.78						
**DR** vs **AMD** **RE&LE** PCT	0.73	0.58	0.81	0.70	0.69	0.32						

Supplementary Table 2: Study 2, green sequence. AUC of ROC, sensitivity, specificity and effect size calculated with different PCT variables comparing HP and AMD, HP and DR, AMD and DR participants. The leftmost column indicates the variables used in AUC calculation using *fitglm* and *perfcurve* functions (Matlab, version R2018b): PCT refers to oscillation period, amplitude, variability and regularity, including FFT power and frequency measured on pupil traces; GPS are 5 global Pupil State variables, and COR refers to variables related to data corrections (see Data Analyses section).

Supplementary Figures

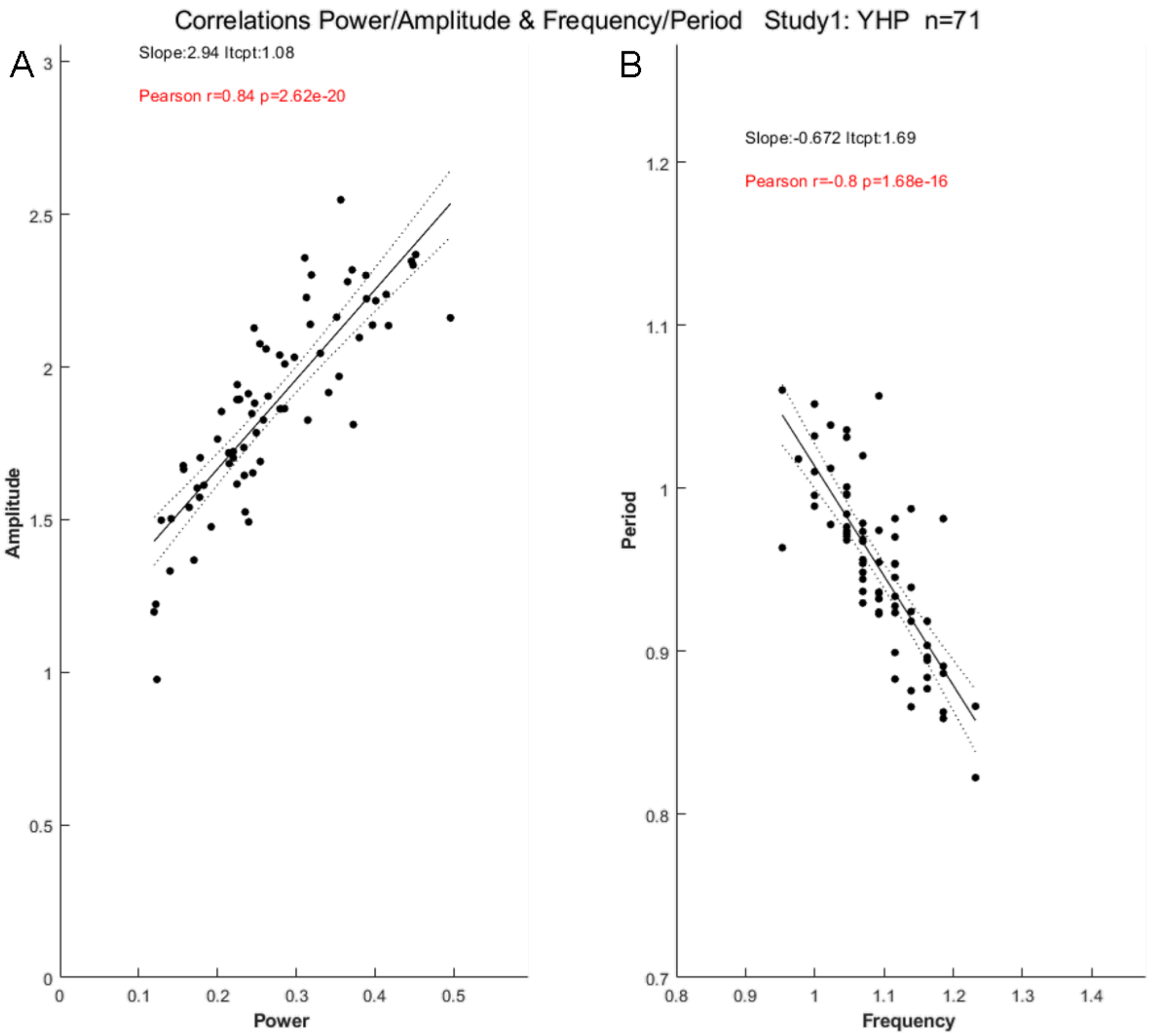

Supplementary Figure 1: A. Correlation between PCT spectral power and amplitude derived from oscillations' peaks and troughs. B. Correlation between PCT spectral frequency and period derived from the timing of oscillations' peaks. Correlation coefficients are high (>0.8), indicating that the extraction of period and amplitude from the maxima and minima of the corrected pupil traces reflect PCT features similar to the frequency and power derived from spectral analyses.

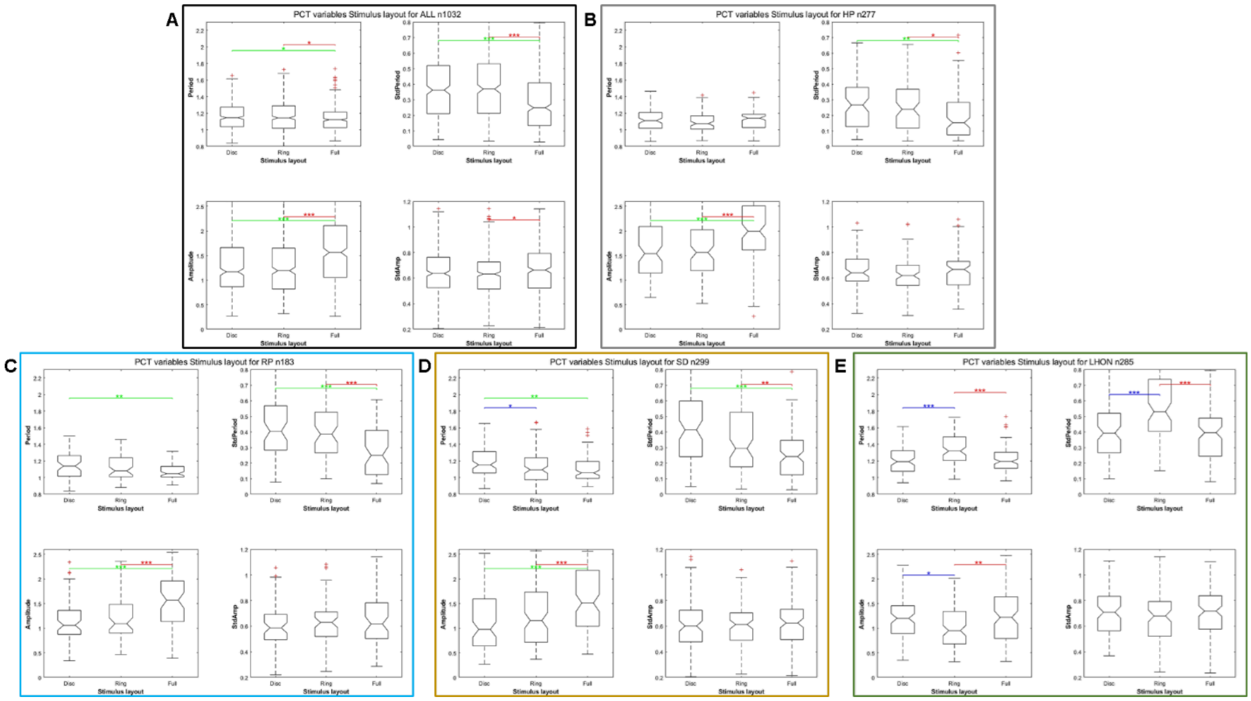

Supplementary Figure 2: Study 3: Distributions of PCT values for each group and for different configurations: Disc, Ring and Full-field. Each of the A-E panels show the PCT values: Top graphics: Period, Regularity. Bottom graphics: Amplitude, std Amplitude. A. Statistics for the whole data set and each PCT variable. B. Same as A for HP subjects. C. Same as A for RP patients. D. Same as A for SD patients. E. Same as A for LHON patients. One can observe that the period depends little on the spatial layout, and that regularity is better, while the Full-field stimulus elicits larger amplitude and less variable responses than the disc or ring stimuli. All configurations elicit oscillations at similar periods except for LHON and RP patients. All patients tend to exhibit more irregular oscillations of variable amplitude, relative to HP.

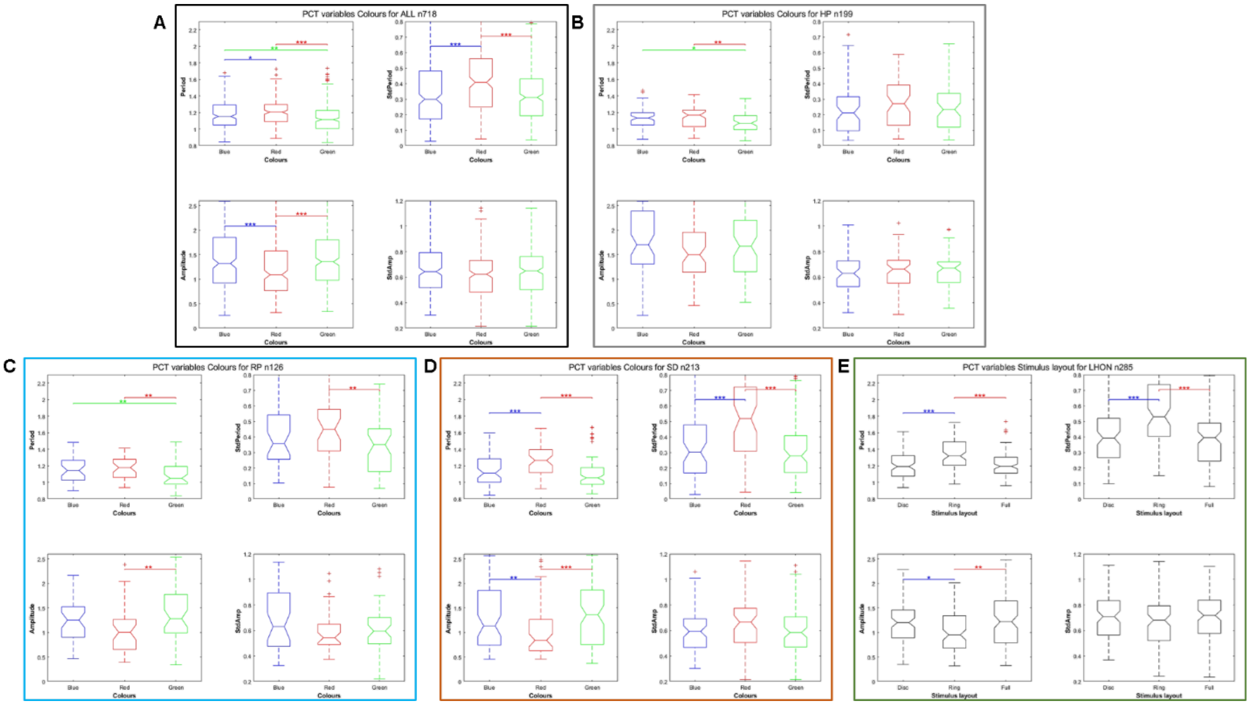

Supplementary Figure 3: Study 3: Distributions of PCT values for each group and for different colours: Blue (blue), Red (red), and Green (green). A-E panels show the PCT values: Top graphics: Period, Regularity. Bottom graphics: Amplitude, std Amplitude. A. Statistics for the whole data set and PCT variables. B. HP subjects. C. RP patients. D. SD patients. E. LHON patients.

The Red stimulus elicits less power and more variable responses than the Blue or Green stimuli, whereas Blue and Green elicit oscillations of similar amplitude. The periods differ slightly between colours, being shorter for the Green than for the Blue and longer for the Red stimulus (A). The values for the Red stimulus are worse for SD and LHON patients. All colours elicit oscillations at similar frequencies except for LHON and SD patients. Patients tends to exhibit more irregular oscillations of more variable amplitude relative to HP.

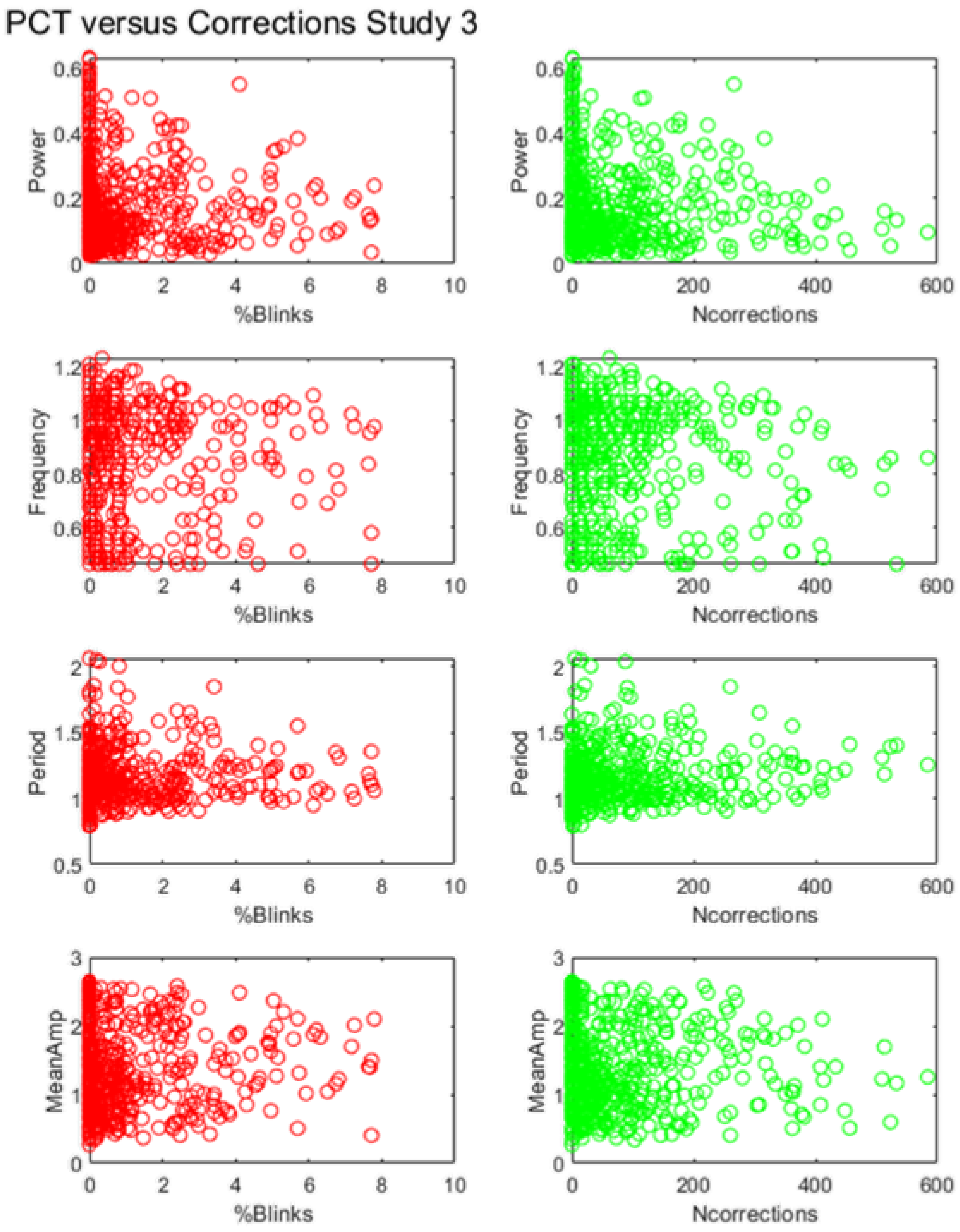

Supplementary Figure 4: Study 2: PCT variables as a function of the number of data corrected for blinks or spurious transients (data corresponding to fast pupil changes incompatible with the sluggish pupil responses). No correlations are observed for any of the PCT variables.

Supplementary Materials: Control Experiment, Effect of Gain

We tested the effect of Gain, the value used to convert Pupil size into luminance, on PCT in a control experiment using a grey disc stimulus identical to that of Study 1. Different Gain values (0.2, 0.5, 1, 2, 3, 5, 8 and 10) were tested with a single observer (one author, 70 years old, no correction) who performed several runs for each gain value on different days. As expected, the different gains changed the mean stimulus luminance, but sustained oscillations were elicited for all gains greater than 1 and less than 10 (Supplementary Figures 4 and 5). With a gain of 10, the product of gain and pupil size (about 30 A.U.) gives values above 255 which is the upper limit of RGB values (white screen). Consequently, pupil constriction reaches its minimum and cannot oscillate. Similarly, a gain below 1 led to low stimulus luminance and a floor effect. For gains between 1 and 5, PCT variables remain within a similar range (oscillation amplitude: 2.2 and 1.9 for gains of 1 and 5; oscillation frequency: 0.86, 0.91, and 0.88 Hz for gains of 1, 2, and 5, respectively), and oscillations are regular over time (Supplementary Figure 4).

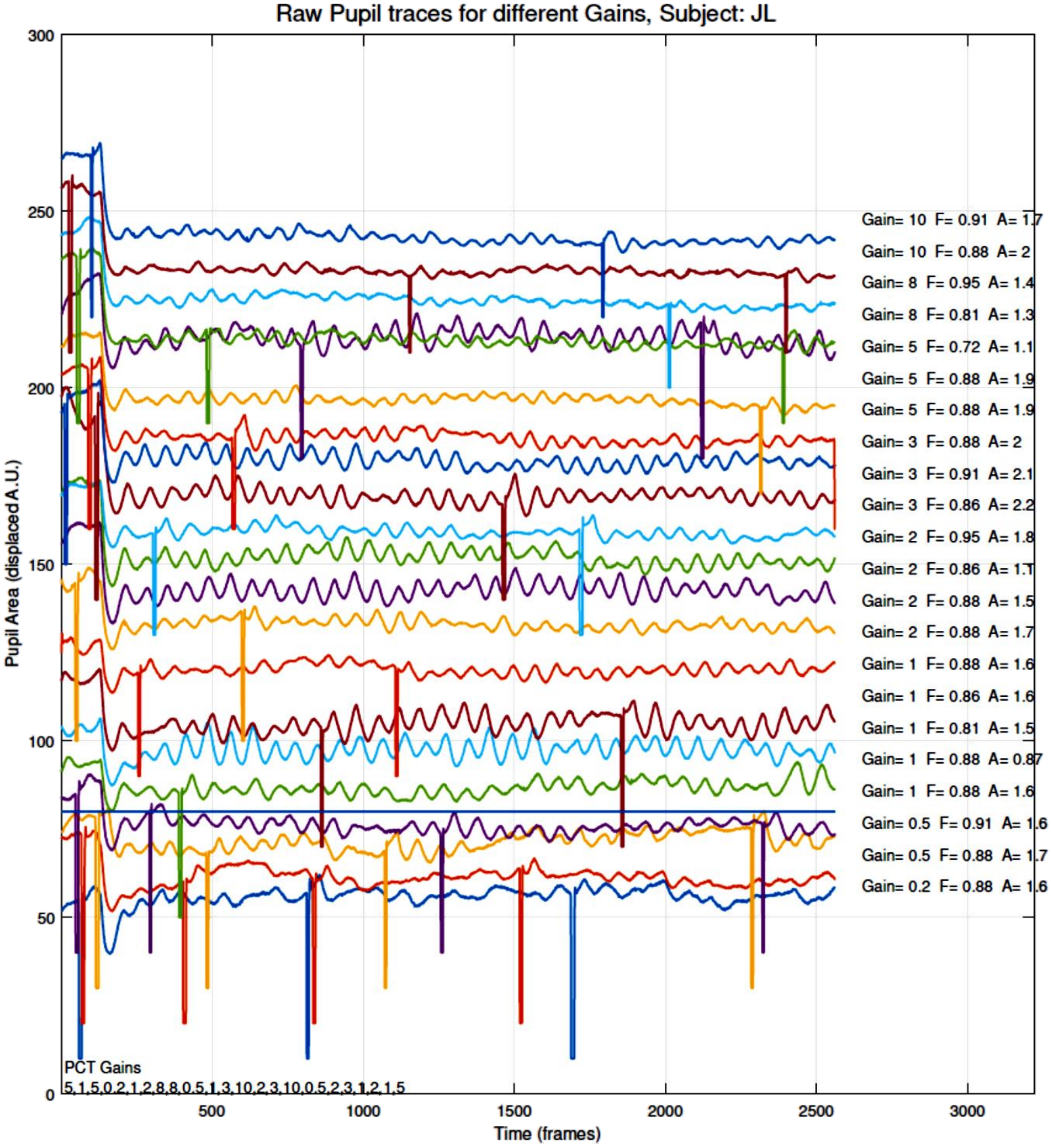

Supplementary Figure 5: Raw traces of pupil oscillations elicited for 45 seconds with different gains used to convert pupil size into luminance (RGB guns values) using the formula:

$$L_{rgb} = \text{Pupil area x Gain.}$$

Data for a single subject JL (co-author, 70 years old). Gains ranged from 0.2 to 10 (0.2, 0.5, 1, 2, 3, 5, 8, 10). Several repetitions with the same gain were performed to evaluate repeatability. In this experiment the eye-tracker delivered pupil area values ranging from 5 to 50. Low gains induced oscillations around low luminance values, while high gains induced oscillations around

high luminance values. A gain of 10 leads to saturation (L_{RGB} luminance around 255) with small and irregular oscillations. Texts in regards of the pupil traces indicate the Gain, the oscillation frequency, F, and the oscillation amplitude, A, for each run. As can be seen, reliable and regular oscillations are found for gains ranging from 1 to 8. Vertical drops are blinks.

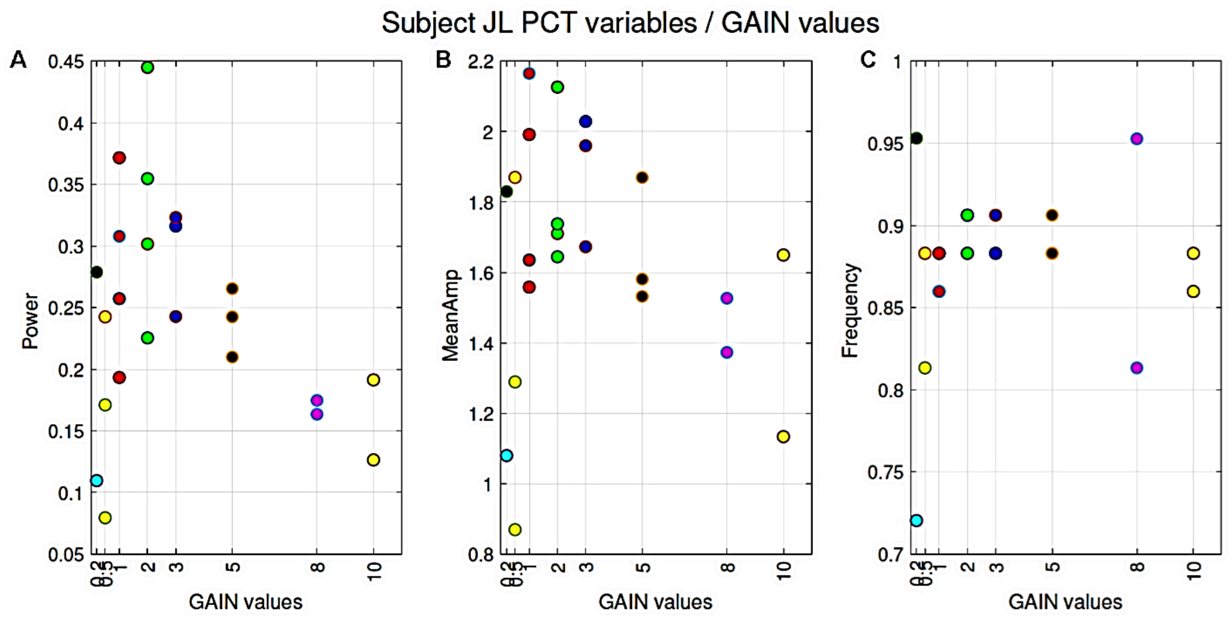

Supplementary Figure 6: Distribution of PCT variables as a function of gain values for a single subject. A. Power as a function of gain. Gains between 1 and 5 elicit the largest power. Values below or above 1 and 5 elicit reduced oscillation power. B. Mean oscillation amplitude appears a band pass function of gain. C. Oscillation frequency is stable for gains between 1 and 5.
